# Determinants of AI Adoption in Saudi Arabian Healthcare Institutions

**DOI:** 10.3390/healthcare14131833

**Published:** 2026-06-24

**Authors:** Saeed Ali Al-Shahrani, Zahyah H. Alharbi, Tahani Alqurashi

**Affiliations:** 1 Department of Management Information Systems, College of Business Administration, King Saud University, Riyadh 12372, Saudi Arabia; 445910844@student.ksu.edu.sa; 2 Department of Data Science, College of Computing, Umm Al-Qura University, Makkah 24381, Saudi Arabia; tmqurashi@uqu.edu.sa

**Keywords:** artificial intelligence in healthcare, AI acceptance, Saudi healthcare, ethical governance, trust in AI

## Abstract

**Background/Objectives:** Artificial Intelligence (AI) integration in healthcare promises improved diagnostic accuracy, patient safety, and operational efficiency. However, AI acceptance among healthcare workers remains limited due to knowledge gaps, risk concerns, and governance challenges, particularly in developing countries like Saudi Arabia, where rapid healthcare modernization faces unique infrastructure, organizational, and cultural challenges. This research investigates the factors influencing AI acceptance among medical practitioners, nurses, administrators, and students in Saudi Arabian hospitals to identify key determinants and barriers to adoption. **Methods:** This cross-sectional study employed an extended Unified Theory of Acceptance and Use of Technology (UTAUT) framework integrated with ethical considerations from the Model for Ethical Assessment and Analysis of AI in Medicine (MEAAM). A structured bilingual questionnaire was administered to 119 healthcare professionals and students across Saudi Arabia, measuring constructs including Awareness and Knowledge, Performance Expectancy, Effort Expectancy, Facilitating Conditions, Social Influence, Trust, Perceived Risk, Ethical Governance, and Price Value. Partial Least Squares Structural Equation Modeling (PLS-SEM) was employed for quantitative analysis, supplemented by thematic analysis of open-ended qualitative responses. **Results:** The PLS-SEM analysis explained 59.8% of variance in behavioral intention to adopt AI (R^2^ = 0.598). Awareness and Knowledge emerged as the strongest predictor (β = +0.505, *p* < 0.001), followed by Performance Expectancy (β = +0.229, *p* < 0.05) and Social Influence (β = +0.123). Perceived Risk functioned as the primary barrier (β = −0.185, *p* < 0.05). Qualitative findings identified infrastructure gaps, regulatory ambiguities, and training deficiencies as major implementation barriers, while emphasizing opportunities in diagnostic accuracy and remote monitoring. **Conclusions:** AI acceptance in Saudi healthcare is primarily driven by knowledge, with perceived usefulness and peer support as secondary facilitators, while safety and accountability concerns remain substantial obstacles. Successful AI integration requires coordinated efforts in education, transparent governance frameworks, and institutional support. This study contributes theoretically by validating extended UTAUT in a non-Western healthcare context and practically by providing evidence-based strategies for sustainable AI adoption that enhance healthcare quality while respecting professional roles and ethical principles.

## 1. Introduction

The integration of artificial intelligence (AI) into healthcare has been widely described as one of the most consequential technological shifts in modern medicine, offering transformative potential in diagnostics, efficiency, and patient care [1]. However, translating this potential into practice requires understanding why healthcare professionals accept or resist AI; international survey evidence demonstrates that factors such as trust, perceived risk, and institutional support are at least as important as the technology itself [2]. Nowhere is this gap between potential and adoption more evident than in Saudi Arabia, where the healthcare sector is undergoing rapid modernization driven by Vision 2030’s health transformation agenda, the National Digital Health Strategy (NDHS 2018–2030), and the Saudi Health Information Exchange (SHIE) initiative, yet faces unique challenges in technical infrastructure, organizational readiness, and cultural considerations [3,4,5,6]. Despite a growing body of descriptive work documenting awareness levels and general attitudes toward AI among Saudi healthcare professionals, the field lacks theoretically grounded, multi-construct empirical analyses that can explain why acceptance remains constrained and identify the relative weight of cognitive, organizational, ethical, and financial determinants in shaping adoption intentions. Without such evidence, interventions risk targeting proximal symptoms while leaving deeper structural barriers unaddressed. The present study addresses this gap by integrating MEAAM-derived ethical governance into an extended UTAUT model and employing PLS-SEM to simultaneously estimate nine determinants of AI acceptance.

Understanding the determinants of AI acceptance among healthcare professionals is critical for translating technological potential into clinical practice, as without such evidence, healthcare institutions and policymakers lack the foundation needed to design targeted and effective interventions. This research aims to provide a detailed study of the degree of acceptance and knowledge towards AI in healthcare among medical personnel and students working in hospitals in Saudi Arabia, with particular focus on the cognitive, organizational, technical, and ethical factors that influence AI adoption decisions. The investigation is grounded in the expanded Unified Theory of Acceptance and Use of Technology (UTAUT) framework and incorporates ethical considerations from the Model for Ethical Assessment and Analysis of AI in Medicine (MEAAM) model. The UTAUT framework identifies four primary factors—performance expectancy, effort expectancy, social influence, and facilitating conditions—as major predictors of behavioral intention and actual usage of innovative technologies [7]. The model also refers to age, gender, experience, and voluntariness of use as moderator variables [8]. However, moderation analysis is outside the scope of this study given the sample size constraints and is reserved for future research with larger samples. The MEAAM model provides a conceptual framework for addressing ethical concerns in AI healthcare systems. It divides thirteen ethical considerations into four pillars—Fair AI, Responsible AI, Explainable AI, and Sustainable AI—and analyzes them through three ethical perspectives (epistemic, normative, and overarching) with the purpose of examining how moral issues influence AI implementation at both operational and systemic levels in healthcare [9].

Despite growing recognition of AI’s potential across health systems globally, adoption in Saudi Arabia still faces a complex network of technical, organizational, ethical, and financial barriers. Recent literature indicates that although most healthcare practitioners in Saudi Arabia are knowledgeable about AI concepts at a general level, their involvement with smart technologies remains minimal [3,4], mainly due to inadequate digital infrastructure, insufficient personnel training, and limited institutional understanding of AI systems. Trust in AI and the ease of its adoption are negatively affected by these gaps, creating major obstructions to the actual use of AI in Saudi hospitals. Technical barriers compound this further: issues include substandard digital health data, limited AI system capabilities, and ongoing challenges in integrating new smart devices into existing clinical workflows. These technical limitations are further exacerbated by administrative and regulatory challenges, including a lack of standardized regulatory rules governing AI use in clinical settings. Moreover, there is an insufficient workforce with the necessary skills in both medicine and technology to implement large-scale projects, and unclear legal provisions regarding AI use make it even more challenging to realize implementation plans. Many healthcare practitioners tend to view AI as a supportive tool for clinical decision-making rather than as a replacement for physicians, with broad consensus favoring human–AI collaboration over complete substitution.

The issues of transparency, accountability, and justice in decision-making through AI have a significant impact on the extent of acceptance and trust in these systems. If decisions are not made transparently, or if the sharing of responsibilities between human and AI agents is unclear, feelings of danger and mistrust emerge, even when technical performance is good. The literature indicates that users’ positive theoretical expectations about AI’s capabilities are not always reflected in their actual behavioral adoption. Without adequate institutional support, capable leadership, and a clear economic rationale for AI projects, practical applications of AI are usually restricted in everyday work, even when there is broad theoretical understanding.

To address these challenges, this study employs a questionnaire tailored to the situation in Saudi Arabia that considers various indicators measuring awareness, acceptance, and the human–AI relationship, as well as the difficulties of technology and organizations related to the actual implementation of systems. Additional measures are used to assess performance expectancy, effort expectancy, trust, perceived risk, and organizational, financial, and training drivers. Together, these factors enable a detailed evaluation of the variables affecting the practical implementation of AI, linking user perceptions to actual behaviors and barriers within institutions.

The objectives of this study are as follows:

- To assess the knowledge of healthcare workers and students about AI applications in the Saudi health system.
- To identify the behavioral intentions and attitudes of people towards medical AI technologies.
- To determine the influence of performance expectancy, trust, and institutional readiness on the intention to adopt AI.
- To present evidence-based suggestions for the facilitation of effective and sustainable adoption policies in the hospitals of Saudi Arabia.

To achieve these objectives, the research is guided by the following research questions:

- RQ1 (AI Awareness and Current Attitudes):

What is the current level of AI awareness, knowledge, and behavioral intention among healthcare professionals in Saudi Arabian hospitals?

- RQ2 (Key Determinants of AI Acceptance):

Which cognitive, organizational, and ethical factors most strongly predict healthcare professionals’ intention to adopt AI systems?

- RQ3 (Role of Risk and Trust):

How do perceived risks, trust, and ethical governance influence AI acceptance in the Saudi healthcare context?

- RQ4 (Barriers and Recommendations):

What are the primary barriers to AI implementation in Saudi hospitals, and what practical strategies would increase acceptance?

## 2. Related Work

The literature on AI adoption in Saudi healthcare reveals varying levels of awareness, readiness, and acceptance among different stakeholder groups, along with persistent technical and organizational challenges that affect implementation. This section examines existing research on awareness and knowledge levels, academic readiness, patient safety implications, nursing perspectives, and the barriers encountered in Saudi healthcare settings.

A cross-sectional survey involving 771 members of the Saudi community found that 42.5% see AI as a good thing in the healthcare sector, 31.8% are neutral, and only 7.5% have a negative opinion [10]. The relationship between AI adoption and past technology use, along with self-confidence in technology, was quite evident [10]. In a study [4] conducted across four hospitals in Riyadh, it was found that although healthcare workers had high functional awareness, their knowledge of AI’s practical applications was minimal. The participants raised concerns about the possibility of losing jobs and about insufficient practical training. These findings reveal a critical gap between general awareness and applied clinical understanding, with direct implications for AI adoption in practice.

A nationwide survey of 1221 medical and health sciences students using the MAIRS-MS scale found that these students exhibited moderate (about 57–62%) readiness in cognitive, skills, ethical, and vision areas. A notable proportion of respondents (44.5%) suggested that AI courses should be compulsory in health education programs [11,12,13]. This indicates that while students demonstrate some preparedness for AI integration, substantial gaps remain in their comprehensive understanding and skill development necessary for effective AI utilization in clinical practice.

In a study conducted at Najran University Hospital [3], a significant positive impact was found, with a coefficient R^2^ of 0.60, indicating that a positive attitude toward AI; along with knowledge, this had a major positive effect on patient safety culture. The attitude factor had a greater influence (B = 0.502) [3,10,14]. This demonstrates the potential benefits of AI acceptance beyond operational efficiency, extending to critical outcomes such as patient safety and quality of care.

Serbaya et al. [5] conducted a qualitative study with 48 registered nurses to identify the enablers and obstacles to AI adoption, including organizational support, comprehensive training, and professional and technical matters. The investigation suggested a Technology Acceptance Model for AI in Nursing (TAM-AIN) framework as a new, locally based hypothetical model. This nursing perspective reveals profession-specific concerns and requirements that differ from those of physicians or administrators, emphasizing the need for tailored approaches to AI implementation across different healthcare roles.

In-depth interview research with 30 practitioners in primary care in an unknown city of Saudi Arabia had found that they faced obstacles like a slow internet connection, bad system interfaces, lack of training, and technical support along with many and rigid smart systems such as Clinical Decision Support Systems (CDSSs) [6,15]. Another study [16] found that CDSSs are still not widely accepted by the people. The reasons were that they have mismatches in technical and functional areas and are also not sufficient. These technical and organizational obstacles represent significant barriers to AI adoption, highlighting the gap between theoretical potential and practical implementation in Saudi healthcare settings.

Multiple studies [16,17] revealed that typical hindrances to the implementation of CDSSs included technical problems, inadequate training, poor integration, and a lack of regulatory standards. The studies also highlighted that these issues were present in other developing countries and that Saudi Arabia’s situation was not unique. This comparative perspective suggests that the challenges faced in Saudi healthcare are part of broader patterns observed in similar contexts, although specific cultural, regulatory, and organizational factors in Saudi Arabia require tailored solutions.

## 3. Methodology

### 3.1. Theoretical Framework

The extended Unified Theory of Acceptance and Use of Technology (UTAUT) [18] is a model that describes how the consumer’s decision of technology adoption is influenced by the interaction of factors such as performance expectancy, effort expectancy, social influence, and the availability of institutional support. This study is based on the UTAUT model for technology acceptance, but it has been updated by adding some main present-day factors, for example, trust, perceived risk, and ethical values (explainable AI, fairness, accountability), which are issues in the AI sector in the healthcare field.

**Figure 1:**
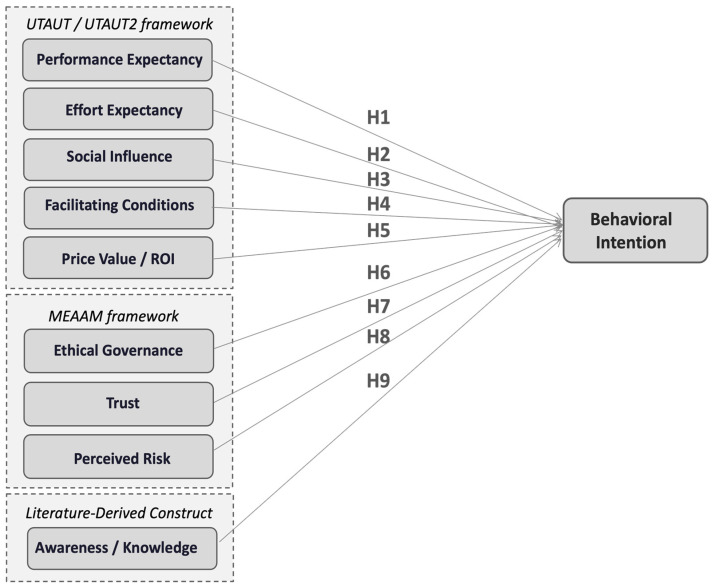
Proposed conceptual research model integrating the extended UTAUT/UTAUT2 and MEAAM frameworks alongside literature-derived construct. Arrows represent hypothesized relationships with Behavioral Intention (H1–H9).

The Model for Ethical Assessment and Analysis of AI in Medicine (MEAAM) [9] provides a structured framework specifically designed to evaluate ethical concerns arising from AI deployment in healthcare settings. The model organizes thirteen ethical considerations into four pillars: Fair AI, which addresses bias prevention and equitable treatment; Responsible AI, which concerns accountability and governance; Explainable AI, which focuses on transparency and interpretability of AI decisions; and Sustainable AI, which examines long-term societal and environmental implications. These pillars are analyzed through three ethical perspectives, namely epistemic, normative, and overarching, to examine how moral issues influence AI implementation at both operational and systemic levels. In the present study, MEAAM informs the analytical framework by providing the theoretical basis for the Ethical Governance construct, capturing healthcare professionals’ expectations for transparency, fairness, and accountability as determinants of AI acceptance. This integration extends the standard UTAUT model beyond purely functional acceptance factors to incorporate the ethical dimensions uniquely relevant to medical AI adoption. Figure 1 presents the proposed conceptual research model, illustrating the hypothesized relationships between the UTAUT/UTAUT2 constructs, the MEAAM-derived Ethical Governance construct, and Behavioral Intention.

The suitability of UTAUT for this context is further supported by a recent systematic review and meta-analysis of healthcare technology adoption, which confirms that performance expectancy consistently emerges as the strongest predictor of behavioral intention across healthcare settings [19].

Adopting the UTAUT framework facilitates understanding of the attitudinal differences among Saudi healthcare professionals, including physicians, nurses, administrators, and students, towards the implementation of AI technologies across diverse regulatory and institutional contexts. Within this framework, ethical considerations, financial factors, managerial support, and training emerge as essential elements that can either facilitate or hinder acceptance.

This theoretical lens enables a reading of the manner in which personal dispositions merge with organizational structures and government policies and understanding the reasons why certain institutions or demographic groups are generally more rapid in the adoption of AI than others—even when the same resources or infrastructure are available.

### 3.2. Research Design

This research employed a cross-sectional quantitative design to investigate the factors influencing healthcare professionals’ acceptance of artificial intelligence (AI) systems in Saudi hospitals. The survey instrument was developed based on the extended UTAUT model [18] and incorporated trust, perceived risk, and ethical considerations from the MEAAM framework [9], along with organizational and financial variables informed by Chen et al. [20].

The study aimed to examine the cognitive, organizational, technical, and ethical factors that influence AI adoption decisions within the context of Saudi healthcare institutions. The adaptive quantitative approach was designed to detect the statistical relationships among variables and provide insights into how organizational and cultural environments affect adoption decisions. This approach was suitable for identifying key determinants and tracing patterns of AI adoption across different institutions and demographic groups in the Saudi healthcare sector.

### 3.3. Data Collection

#### 3.3.1. Sampling Procedures

A purposive sampling strategy was used to target doctors, nurses, administrators, and students with direct experience or interest in AI applications in healthcare. Participants were recruited from both public and private sector institutions. The questionnaire was distributed online through hospital networks, medical associations, and academic platforms. Snowball sampling was employed to broaden coverage and ensure sectoral representation, whereby initial participants were encouraged to share the survey with colleagues from other specialties or hospitals. This approach achieved a diverse sample from various regions and institutions across Saudi Arabia. However, this approach may have favored participants with above-average AI interest, potentially overestimating acceptance levels relative to the broader Saudi healthcare workforce; this limitation is acknowledged in Section 5.5.

The minimum sample size was determined using G*Power (Version 3.1.9.7). Based on the linear regression model (fixed model, R^2^ deviation from 0), with the medium effect size (f^2^ = 0.15), the significance level of 0.05, and the statistical power of 0.80. The model had a total of nine predictors. The analysis results revealed that a minimum sample size of 114 respondents is required to reliably detect the results. The study has a sample size of 119, exceeding the required 114.

The sample included doctors, nurses, administrators, and students in medicine and health sciences, representing both genders and varying levels of experience in government, private, and teaching hospitals. Data confidentiality was maintained through coded identifiers, and all identifying information was removed from the databases. Data collection procedures complied with the ethical standards for medical research established by King Saud University. Informed consent was obtained from all participants prior to data collection.

#### 3.3.2. Survey Instrument

The questionnaire was developed through multiple stages. First, a review of literature and acceptance models (extended UTAUT [8,18,21], MEAAM [9], and Chen et al.’s model [20]) was conducted to identify 25–30 core items. Table 1 presents the measurement constructs, number of items, and source for each construct used in the final instrument. Second, specialists in medical AI and questionnaire design reviewed the instrument. Third, two rounds of pilot testing were conducted with a small sample to assess item clarity and completion time; items that were found to be ambiguous or unclear were reworded to ensure comprehension across diverse healthcare roles, and the survey completion time was confirmed to be within an acceptable range prior to full deployment. A medical AI expert reviewed the final instrument to confirm that all items aligned with established measurement standards for the constructs of interest. To prevent duplicate or multiple submissions, the survey platform was configured to allow only one response per email address, ensuring the integrity of the dataset.

The final survey included demographic variables (age, gender, profession, years of experience, type of institution); acceptance items measured on a 5-point Likert scale covering awareness, performance expectancy, trust, perceived risk, ethical considerations, and organizational factors; and open-ended questions exploring participants’ perspectives on challenges of and opportunities for AI in Saudi hospitals.

The survey was distributed electronically via email and official healthcare institution platforms. Participants received an electronic consent form explaining the study’s purpose, confidentiality protections, and withdrawal rights. Survey completion required approximately 10–12 min. Responses were verified for completeness before analysis. All data were collected electronically and stored in encrypted, password-protected files with restricted access limited to the research team, in compliance with Saudi healthcare research ethics guidelines.

### 3.4. Data Analysis

#### 3.4.1. Quantitative Analysis

Descriptive statistics including frequencies, percentages, means, and standard deviations were computed for sample characteristics and survey items. Instrument validity and reliability were assessed using Cronbach’s alpha, Composite Reliability (CR), and Average Variance Extracted (AVE). PLS-SEM was selected over covariance-based SEM given the study’s modest sample size (N = 119), its exploratory and predictive orientation, and the complexity of the ten-construct model. All analyses were conducted using SmartPLS 4 [20]. Ethical Governance (EG1–EG3) was operationalized as a composite indicator, with items averaged into a single standardized score prior to PLS-SEM estimation, following established composite measurement practice in prediction-oriented PLS-SEM models [22]. Behavioral Intention was measured using a single item (BI3: ‘Worries that AI might replace human experts reduce my willingness to use AI’), which was selected as the most direct measure of adoption intention within the instrument. BI1 and BI2 reflect attitudes toward human-AI collaboration and framing of AI as an assistant rather than a replacement—constructs conceptually distinct from adoption intention, and were therefore retained in the survey for descriptive analysis but excluded from the structural model. The absence of internal consistency metrics for both constructs in the table below reflects these measurement decisions.

Although the original UTAUT framework includes age, gender, and experience as moderating variables, moderation analysis was not conducted due to insufficient statistical power at N = 119 for reliable detection of interaction effects and the inability to form balanced subgroups without severely reducing cell sizes. The moderating role of demographic variables is reserved for future research with larger samples.

#### 3.4.2. Qualitative Analysis

Open-ended responses from the same 119 survey participants were analyzed using thematic analysis [23] to identify perspectives on AI implementation challenges, opportunities, and recommendations. The open-ended questions were embedded within the survey instrument and completed by the same participants who responded to the quantitative items; no separate interviews were conducted. Thematic analysis was performed using NVivo (Version 15) qualitative data analysis software. Two authors independently coded the responses to ensure analytical rigor, with emerging themes discussed and reconciled through consensus. The coding process followed an inductive approach, allowing themes to emerge from the data rather than being imposed from a predetermined framework. Identified themes were organized into three categories corresponding to the three open-ended questions: barriers to implementation, opportunities for healthcare improvement, and recommendations for increasing acceptance.

#### 3.4.3. Validity and Reliability

Multiple approaches were used to ensure validity and reliability. Triangulation was applied by comparing findings from quantitative and qualitative analyses against existing literature and previous field studies. Thematic saturation was pursued in the qualitative data. Where possible, field experts were consulted to review and validate qualitative findings.

The analysis combined quantitative survey results with qualitative insights to provide a comprehensive understanding of AI acceptance determinants, implementation barriers, and the influence of organizational and cultural factors in Saudi healthcare settings. The questionnaire included a statement indicating its academic research purpose. Each of the ten survey sections was analyzed individually for descriptive statistics, reliability, and validity before conducting an overall PLS-SEM analysis to examine relationships among the theoretical variables.

## 4. Results

This section presents the findings from the survey of 119 healthcare professionals in Saudi Arabia. The results are organized into five parts: sample characteristics, consolidated reliability and validity metrics, construct-level response distributions, structural model results from PLS-SEM analysis, and qualitative insights from open-ended questions.

### 4.1. Sample Characteristics

A total of 119 completed questionnaires were obtained from healthcare professionals and students across Saudi Arabia, exceeding the minimum required sample size of 114 as determined by the a priori power analysis. Table 2 presents the demographic characteristics of the sample. The sample demonstrated diversity across gender, age groups, professional roles, institutional affiliations, and experience levels. Professional roles included nurses, administrators, physicians, students, and other healthcare staff representing government hospitals, private hospitals, and other healthcare facilities. Experience levels ranged from less than one year to more than 15 years, providing representation across career stages and institutional settings.

### 4.2. Construct Reliability and Validity

All survey items were measured on a 5-point Likert scale (1 = Strongly Disagree, 5 = Strongly Agree) and assigned to theoretical constructs based on the extended UTAUT framework and MEAAM ethical considerations. Table 3 presents descriptive statistics and psychometric properties for each construct. Cronbach’s alpha (α) assessed internal consistency, with values above 0.70 considered acceptable. Composite Reliability (CR) and Average Variance Extracted (AVE) were used to evaluate convergent validity, with thresholds of 0.70 and 0.50, respectively.

Most constructs demonstrated acceptable to good reliability and convergent validity. Awareness/Knowledge showed excellent psychometric properties (α = 0.818, AVE = 0.849, CR = 0.918). Facilitating Conditions (α = 0.799, AVE = 0.507, CR = 0.861), Trust (α = 0.704, AVE = 0.635, CR = 0.837), Perceived Risk (α = 0.732, AVE = 0.791, CR = 0.883), and Price Value (α = 0.638, AVE = 0.735, CR = 0.847) all met or approached acceptable thresholds. Social Influence showed borderline alpha (α = 0.587) but adequate AVE and CR values.

Performance Expectancy and Effort Expectancy exhibited measurement issues. Performance Expectancy showed low internal consistency (α = 0.382) and below-threshold AVE (0.462). However, the CR approached 0.70 and AVE approached 0.50. Effort Expectancy demonstrated similar problems (α = 0.260, AVE = 0.421). The reliability and validity for Effort Expectancy were low.

While multi-item measures are generally preferred, the use of a limited number of items is considered acceptable in PLS-SEM under certain conditions. PLS-SEM is primarily prediction-oriented and places greater emphasis on indicator reliability and construct validity rather than the number of items per se. Prior research suggests that constructs with two indicators can be retained if they demonstrate adequate internal consistency and validity [22]. Furthermore, methodological literature acknowledges that two-item constructs may be appropriate when the construct domain is conceptually narrow, indicators are highly correlated, and reliability measures meet recommended thresholds [22,24]. Consistent with the PLS-SEM literature, constructs with two indicators can be considered acceptable when they demonstrate adequate reliability and validity [24,25]. For Effort Expectancy, the values were marginally below the recommended threshold. However, the construct was retained as it is conceptually important and grounded in prior literature, making its inclusion essential for model completeness [25]. The composite reliability is close to the acceptable threshold of 0.70, and AVE is over 0.40 with significant outer loadings (*p* < 0.05) [26]. Additionally, Fornell and Larcker [27] suggested that a construct’s convergent validity is still adequate if it has an AVE less than 0.50, but a CR above 0.60.

Discriminant validity was assessed using the Fornell–Larcker criterion and HTMT ratios. Analysis revealed adequate discriminant validity for most constructs. However, Performance Expectancy and Effort Expectancy exhibited measurement issues: two Fornell–Larcker violations and several HTMT values exceeding 0.85 were identified among PE, EE, SI, and Trust.

### 4.3. Construct-Level Response Patterns

This section summarizes response distributions for key items within each construct. Complete item-level statistics are available in Appendix A; representative patterns are highlighted here to illustrate how respondents perceived different aspects of AI in healthcare.

#### 4.3.1. Awareness and Knowledge

Respondents reported moderate awareness of AI applications in healthcare (M = 3.79, SD = 1.07). The majority expressed familiarity with AI in general health contexts (65.6% agree/strongly agree), medical image analysis (63.9% agree/strongly agree), and clinical decision support systems (65.6% agree/strongly agree). Awareness was slightly lower for AI-powered remote monitoring (52.1% agree/strongly agree) and highest for virtual health assistants or chatbots (64.7% agree/strongly agree). Overall, responses indicated baseline familiarity with AI concepts but suggested room for deepening our understanding of specific clinical applications.

#### 4.3.2. Performance Expectancy

Performance expectancy scores were relatively high (M = 3.92, SD = 1.00), indicating positive perceptions of AI’s potential benefits. Strong majorities believed AI could improve diagnostic precision (74.8% agree/strongly agree), reduce human errors (74.0% agree/strongly agree), support accurate decision-making (64.7% agree/strongly agree), and enhance resource allocation through big data analytics (75.7% agree/strongly agree). These patterns suggested that healthcare professionals recognized AI’s value proposition in clinical contexts, though a notable minority (19.3–28.6%) remained neutral, indicating incomplete conviction about performance benefits.

#### 4.3.3. Effort Expectancy and Facilitating Conditions

Effort Expectancy responses showed moderate confidence in learning AI systems (M = 3.98, SD = 0.96), with 73.2% agreeing or strongly agreeing that AI-based healthcare systems would be easy to learn. Confidence was slightly lower regarding handling AI-assisted surgical robots even with training (63.9% agree/strongly agree).

Facilitating Conditions revealed more substantial concerns (M = 3.90, SD = 0.98). Only 43.7% agreed or strongly agreed that their hospital provided sufficient AI training and support, while 28.5% disagreed or strongly disagreed. Perceptions of IT infrastructure adequacy were slightly better (57.2% agree/strongly agree), though 31.1% remained neutral and 11.8% disagreed.

#### 4.3.4. Social Influence

Social Influence scores were among the highest observed (M = 4.02, SD = 0.93). Strong majorities indicated that colleague encouragement increased adoption willingness (78.1% agree/strongly agree) and that senior management support was essential for successful implementation (84.9% agree/strongly agree). Respondents also recognized the influence of social perceptions, including patient opinions and public acceptance (70.6% agree/strongly agree). These patterns demonstrated that normative pressures and institutional endorsement substantially shaped individual attitudes toward AI adoption.

#### 4.3.5. Trust and Perceived Risk

Trust in AI recommendations was moderately high (M = 4.12, SD = 0.85), with 67.2% agreeing or strongly agreeing they trusted AI-powered clinical decision-making outputs. However, 29.4% remained neutral, suggesting incomplete confidence. Notably, respondents recognized AI’s potential to enhance data security through encryption and access controls (71.4% agree/strongly agree).

Despite generally positive trust, Perceived Risk remained elevated (M = 3.92, SD = 1.02). Strong majorities expressed concern about relying on AI in high-risk cases such as cancer diagnosis or major surgery (68.1% agree/strongly agree), worried about patient data privacy and confidentiality (66.4% agree/strongly agree), and were concerned about legal accountability for AI-related medical errors (73.1% agree/strongly agree). Concerns about AI’s ability to detect and prevent human errors were also substantial (57.2% agree/strongly agree). These patterns revealed that even healthcare professionals with positive attitudes toward AI maintained significant reservations about safety, privacy, and liability issues.

#### 4.3.6. Ethical Governance

Ethical Governance expectations were very high (M = 4.21, SD = 0.83). Overwhelming majorities believed that medical AI systems should be explainable and transparent (80.7% agree/strongly agree), that fairness and lack of bias were essential for acceptance (79.0% agree/strongly agree), and that clear ethical guidelines and accountability structures increased trust (82.3% agree/strongly agree). These strong endorsements indicated that ethical considerations were not peripheral concerns but central requirements for AI acceptance in Saudi healthcare.

#### 4.3.7. Price Value and ROI

Financial considerations showed more varied patterns (M = 3.73, SD = 1.05). Most respondents agreed that positive ROI should be demonstrated before adoption (71.4% agree/strongly agree), and that financial incentives or government support would encourage acceptance (71.5% agree/strongly agree). However, opinions were mixed regarding whether insufficient funding currently limited AI adoption, with 45.4% agreeing, 34.5% neutral, and 20.2% disagreeing.

#### 4.3.8. Behavioral Intention and Human-AI Collaboration

Behavioral Intention was moderate (M = 3.48, SD = 1.23), indicating cautious readiness to adopt AI. Strong majorities endorsed AI as an assistant rather than replacement (86.6% agree/strongly agree) and believed that combining AI with healthcare professionals was key to success (85.7% agree/strongly agree). However, 53.8% agreed or strongly agreed that worries about AI replacing human experts reduced their willingness to use AI.

### 4.4. PLS-SEM Structural Model Results

Partial Least Squares Structural Equation Modeling (PLS-SEM) was performed to examine relationships among theoretical constructs and their influence on Behavioral Intention. The model was estimated using component-based PLS with standardized composite scores. Table 4 reports standardized path coefficients, standard errors, t-values, and significance levels.

As shown in Table 4, Awareness/Knowledge showed the strongest positive coefficient (β = +0.505, *p* < 0.001), followed by Performance Expectancy (β = +0.229, *p* < 0.05) and Social Influence (β = +0.123). Ethical Governance (β = +0.094) and Facilitating Conditions (β = +0.077) showed smaller positive coefficients. Trust showed a near-zero coefficient (β = +0.019). Perceived Risk showed a negative coefficient (β = −0.185, *p* < 0.05), while Effort Expectancy (β = −0.083) and Price Value/ROI (β = −0.003) showed small negative coefficients. The model explained 59.8% of the variance in Behavioral Intention (R^2^ = 0.598). Three paths were statistically significant at conventional thresholds. Awareness/Knowledge (β = +0.505, *p* < 0.001), Performance Expectancy (β = +0.229, *p* < 0.05), and Perceived Risk (β = −0.185, *p* < 0.05) showed significant coefficients.

Social Influence (β = +0.123), Ethical Governance (β = +0.094), and Facilitating Conditions (β = +0.077) showed positive coefficients that did not reach conventional significance thresholds (*p* > 0.05). Trust (β = +0.019), Price Value (β = −0.003), and Effort Expectancy (β = −0.083) showed coefficients near zero or small negative values.

### 4.5. Qualitative Insights from Open-Ended Responses

Three open-ended questions elicited qualitative perspectives on barriers, opportunities, and recommendations for AI adoption. Response rates were 69 (58.0%) for barriers, 60 (50.4%) for opportunities, and 54 (45.4%) for recommendations. Responses were analyzed thematically, with representative themes summarized below.

#### 4.5.1. Barriers to Implementation

The most frequently mentioned barriers fell into four categories. Infrastructure and technical limitations included inadequate IT systems, poor internet connectivity, lack of interoperability between AI systems and existing hospital software, and insufficient technical support. Representative quotes included: “Our hospital systems are not ready for AI integration—we have basic problems with internet speed and system compatibility” and “There is no technical support team that understands AI systems.”

Governance and regulatory gaps encompassed unclear legal liability for AI errors, absence of national AI ethics guidelines for healthcare, lack of regulatory oversight, and concerns about accountability when AI recommendations led to adverse outcomes. Participants noted: “Who is responsible if AI makes a diagnostic error—the doctor, the hospital, or the technology company?” and “We need clear laws and regulations before we can use AI clinically.”

Training and knowledge deficiencies were mentioned frequently, including insufficient training programs, limited opportunities for hands-on experience with AI systems, lack of educational materials in Arabic, and absence of AI content in medical and nursing curricula. One respondent stated: “We hear about AI, but we never see it or use it—how can we trust something we don’t understand?”

Cultural and professional concerns included fears of job displacement, resistance to changing established clinical workflows, concerns about AI replacing human judgment, and skepticism about technology’s role in patient care. Several respondents expressed: “Healthcare is about human connection—AI might make it too mechanical” and “There is worry that AI will reduce the need for healthcare workers.”

#### 4.5.2. Opportunities for Healthcare Improvement

Respondents identified numerous potential benefits. Diagnostic accuracy and clinical decision support were most frequently mentioned, including improved detection of abnormalities in medical imaging, reduced diagnostic errors through AI second opinions, pattern recognition in complex cases, and support for evidence-based treatment decisions. One physician noted: “AI could help us catch things we might miss, especially in radiology—it acts as a safety net.”

Efficiency and resource optimization opportunities included automation of routine documentation tasks, faster processing of test results, more efficient allocation of hospital resources based on predictive analytics, and reduced waiting times for patients. Administrators emphasized: “AI could help us manage resources better and reduce waste.”

Enhanced patient monitoring and telemedicine capabilities included remote patient monitoring systems using AI, expansion of telehealth services to rural areas, early warning systems for patient deterioration, and continuous monitoring of chronic disease patients. A nurse commented: “AI monitoring could help us track more patients and catch problems early.”

Data-driven insights included analysis of large datasets to identify treatment patterns, quality improvement through analysis of clinical outcomes, personalized treatment recommendations based on patient data, and research support through pattern identification in medical records.

#### 4.5.3. Recommendations for Increasing Acceptance

Participants provided actionable recommendations across multiple domains. Education and training recommendations emphasized comprehensive training programs for all staff levels, hands-on demonstrations and pilot programs before full implementation, integration of AI education into medical and nursing curricula, Arabic-language training materials and resources, and ongoing professional development as AI systems evolve.

Governance and policy recommendations included establishment of national AI ethics guidelines for healthcare, clear legal frameworks defining liability and accountability, transparent oversight mechanisms, regulatory standards for AI system approval and monitoring, and patient consent protocols for AI-assisted care.

Communication and framing strategies stressed the importance of emphasizing AI as a clinical assistant, not a replacement, sharing success stories from early adopters, using culturally appropriate messaging in Arabic, engaging clinical champions to promote adoption, and maintaining transparent communication about AI limitations and risks.

Implementation strategies included phased rollout starting with low-risk applications, pilot programs to demonstrate benefits and identify problems, strong leadership commitment and visible support from management, dedicated technical support teams, and evaluation frameworks to monitor outcomes and adjust implementation.

## 5. Discussion

This study investigated the factors influencing AI acceptance among healthcare professionals in Saudi Arabia using an extended UTAUT framework integrated with ethical considerations from the MEAAM model. The following subsections discuss the main findings, their theoretical and practical implications, implementation barriers and opportunities, methodological considerations, and directions for future research.

### 5.1. Key Determinants of AI Acceptance

#### 5.1.1. Knowledge as the Strongest Predictor

The finding that Awareness and Knowledge was the single strongest predictor of behavioral intention represented the most significant result of this study. As reported in Section 4.2, the construct demonstrated excellent psychometric properties, indicating that the items cohesively measured AI familiarity. This effect was substantially larger than all other factors combined, indicating that comprehension of AI capabilities, applications, and limitations formed the foundation for acceptance. Healthcare professionals who understood how AI functioned in clinical contexts, such as medical image analysis, clinical decision support systems, and remote patient monitoring, were far more likely to express intention to use these technologies than those with limited knowledge.

This finding aligned with Chen et al. [20], who identified knowledge gaps as a primary barrier to AI acceptance among physicians and medical students globally. However, the magnitude of the knowledge effect in this study (β = 0.505) exceeded typical UTAUT studies, where performance expectancy or facilitating conditions often emerge as strongest predictors [18]. This suggested that in the early stages of AI adoption in Saudi healthcare, cognitive readiness—simply knowing what AI is and what it can do—outweighed perceptions of usefulness or ease of use. This pattern was consistent with technology adoption in contexts where innovations are novel and unfamiliar [20].

The qualitative data supported this interpretation. Participants repeatedly mentioned insufficient training and limited exposure to AI systems as barriers to adoption. Many respondents expressed interest in hands-on demonstrations, case-based learning, and opportunities to observe AI systems in practice before committing to their use. This indicated that knowledge gaps were not merely about lacking factual information but reflected a deeper need for experiential understanding and confidence-building through direct engagement with AI technologies.

The practical implication was clear: education and training programs represented the highest-leverage intervention for increasing AI acceptance in Saudi hospitals. Unlike organizational infrastructure or policy frameworks, which required substantial time and investment to develop, targeted education initiatives could produce relatively rapid gains in acceptance by addressing the single most important determinant identified in this study.

#### 5.1.2. Performance Expectancy and Social Influence

Performance Expectancy emerged as the second strongest predictor of acceptance, while Social Influence showed a positive but non-significant association. Performance Expectancy measured the extent to which healthcare professionals believed AI would improve diagnostic accuracy, reduce errors, enhance decision-making, and increase efficiency in resource allocation. Despite lower reliability, indicating measurement overlap among items, the significant positive path coefficient suggested that perceived usefulness remained an important driver of acceptance. However, this finding should be interpreted cautiously given the construct’s low internal consistency, which may introduce instability in the path estimate.

This result was consistent with core UTAUT predictions [9] and with findings from Al-Somali [28], who reported that perceived benefits of AI-powered systems influenced adoption intentions in the Saudi context. However, the relatively modest effect size suggested that believing AI is useful was insufficient for adoption if foundational understanding was lacking. This hierarchical pattern—where higher knowledge scores were associated with greater recognition of benefits—differentiated early-stage adoption contexts from mature technology environments where users already possess baseline familiarity.

Social Influence reflected the impact of peer encouragement, management support, and broader social perceptions on individual adoption intentions. Although the path coefficient did not reach statistical significance (β = +0.123, *p* = 0.168), the positive direction was consistent with UTAUT predictions, and qualitative responses consistently highlighted peer encouragement and management endorsement as contextually important factors. Participants noted that visible endorsement from clinical leaders, successful implementation stories shared among colleagues, and institutional commitment to AI initiatives created normative pressures that may facilitate acceptance. This non-significant result may reflect insufficient statistical power at N = 119 or multicollinearity with related constructs, and warrants re-examination in larger samples.

The combined effect of Performance Expectancy and Social Influence approached the magnitude of the knowledge effect, suggesting that once baseline awareness was established, demonstrating tangible benefits and mobilizing social support became critical for translating positive attitudes into actual adoption. This finding aligned with diffusion of innovation theory, where peer influence and observable advantages accelerate technology uptake after initial awareness is achieved.

#### 5.1.3. The Barrier of Perceived Risk

Perceived Risk emerged as the most substantial negative predictor of behavioral intention, indicating that concerns about privacy, safety, liability, and system errors significantly reduced willingness to adopt AI even among respondents with high awareness and positive attitudes toward the technology. The construct demonstrated strong reliability and convergent validity, suggesting that risk perceptions were coherent and salient among the sampled healthcare professionals.

Specific concerns identified through both quantitative items and qualitative responses included worries about relying on AI in high-risk clinical scenarios (such as cancer diagnosis or major surgery), fears about patient data privacy and confidentiality breaches, uncertainty about legal accountability for AI-related medical errors, and doubts about AI’s ability to detect and prevent human errors. These concerns were not trivial anxieties but reflected legitimate uncertainties in a context where regulatory frameworks, liability structures, and oversight mechanisms for medical AI remained underdeveloped.

Interestingly, despite high mean scores on Trust (M = 4.12), indicating generally positive attitudes toward AI reliability, Perceived Risk remained elevated (M = 3.92) and exerted significant negative influence on intention. The paradox of high trust coexisting with high risk perception suggested that healthcare professionals distinguished between trusting AI’s technical capabilities in principle and accepting the risks associated with actual clinical deployment. The qualitative data clarified this distinction: respondents acknowledged AI’s potential to enhance data security and reduce errors but simultaneously worried about accountability gaps, privacy vulnerabilities, and the consequences of system failures. Theoretically, this finding implies that Trust and Perceived Risk are not simply mirror constructs. Rather, Trust may reflect dispositional or general confidence in AI as a technology category, whereas Perceived Risk captures context-specific concerns about deployment in high-stakes clinical environments, a distinction that separates general confidence appraisals from specific threat appraisals. In the Saudi healthcare context where regulatory frameworks and liability structures for medical AI remain underdeveloped, even professionals who express general trust in AI cannot resolve the institutional uncertainties that elevate perceived risk. This suggests that interventions aimed at increasing trust alone (such as demonstrating AI accuracy) may be insufficient without parallel reductions in structural risk through governance reform and accountability mechanisms.

This finding diverged somewhat from studies in more mature AI adoption contexts, where trust typically correlates more strongly with reduced risk perception. In the Saudi healthcare setting, the absence of clear governance frameworks, liability clarifications, and demonstrated safety records meant that even professionals who viewed AI favorably remained appropriately cautious about implementation. This result underscored the importance of transparent governance, explicit accountability structures, and robust privacy protections as prerequisites for overcoming risk-related barriers to adoption.

### 5.2. Secondary Factors: Enabling and Limiting Adoption

#### 5.2.1. Ethical Governance and Facilitating Conditions

Ethical Governance and Facilitating Conditions showed positive but non-significant path coefficients, suggesting directionally consistent but statistically unconfirmed associations with behavioral intention. Ethical Governance measured expectations for transparency, fairness, bias prevention, and clear accountability frameworks in AI systems. The positive path coefficient indicated that healthcare professionals were more willing to adopt AI when they believed strong ethical safeguards were in place. Qualitative responses reinforced this finding, with participants emphasizing the need for explainable AI, guidelines to prevent algorithmic bias, and clear ethical oversight structures.

The relatively small direct effect of Ethical Governance does not imply low importance. Rather, this effect may in part reflect indirect pathways through Trust and Perceived Risk; however, formal mediation analysis was not conducted in this study, and such indirect effects remain a hypothesis to be tested in future research using bootstrapped confidence intervals. Transparent governance frameworks increased trust in AI systems by providing assurance that decisions would be accountable and fair while simultaneously reducing perceived risks by clarifying liability and establishing oversight mechanisms. This theoretically proposed indirect role offers a plausible explanation for why Ethical Governance emerged repeatedly in qualitative recommendations as essential for acceptance, though this pathway was not formally tested and remains a hypothesis for future research.

Facilitating Conditions measured organizational readiness, including IT infrastructure, training availability, technical support, and system interoperability. The positive effect confirmed UTAUT predictions that institutional support enables adoption. However, the small magnitude suggested that facilitating conditions alone were insufficient to drive acceptance.

Descriptive statistics revealed substantial concerns about organizational readiness. Qualitative insights clarified this pattern: participants noted that even when infrastructure existed, adoption remained limited without corresponding education, leadership endorsement, and demonstrated benefits. This indicated that organizational enablers were necessary but not sufficient conditions for adoption; they removed barriers but did not actively motivate use in the absence of knowledge and perceived value.

#### 5.2.2. Non-Significant Factors: Trust, Price Value, and Effort Expectancy

Trust (β = +0.019), Price Value (β = −0.003), and Effort Expectancy (β = −0.083) showed negligible or counterintuitive effects. The near-zero direct coefficient for Trust is theoretically important and warrants careful interpretation. Despite the conceptual prominence of Trust in the AI acceptance literature, its near-zero direct coefficient likely reflects shared variance with Performance Expectancy and Ethical Governance, all of which capture different dimensions of confidence in AI systems.

Trust in AI recommendations was moderately high (M = 4.12, SD = 0.85), with 67.2% agreeing or strongly agreeing that they trusted AI-powered clinical decision-making outputs. However, 29.4% remained neutral, suggesting incomplete confidence. This paradox—high average trust coexisting with a near-zero path coefficient—can be explained through two complementary mechanisms: collinearity with related constructs and indirect effects through Perceived Risk.

Trust, Performance Expectancy, and Ethical Governance share conceptual overlap as they all capture different facets of confidence in AI systems. When entered simultaneously into the structural model, their shared variance is distributed across the three paths, attenuating the unique direct contribution of Trust. Additionally, Trust may operate primarily as an indirect antecedent of behavioral intention through its mediating relationship with Perceived Risk: professionals who trust AI systems tend to perceive a lower risk, and it is that reduced risk perception—rather than trust itself—that drives adoption intention. In the Saudi healthcare context, where regulatory frameworks and liability structures for medical AI remain underdeveloped, even professionals who express general trust in AI cannot resolve the institutional uncertainties that elevate perceived risk. This suggests that interventions aimed at increasing trust alone may be insufficient without parallel reductions in structural risk through governance reform. Future research should test this mediated pathway explicitly.

The negligible Price Value coefficient (β = −0.003) suggested that economic considerations operated at institutional rather than individual levels. Financial considerations showed varied patterns (M = 3.73, SD = 1.05), with most respondents agreeing that positive ROI should be demonstrated (71.4% agree/strongly agree) but there were mixed opinions on whether funding currently limited adoption (45.4% agreeing, 34.5% neutral, 20.2% disagreeing). This variability suggested that financial viability was relevant for organizational adoption decisions but did not influence individual practitioners’ willingness to use AI once systems were implemented.

The negative Effort Expectancy coefficient (β = −0.083) was likely a measurement artifact given the construct’s very low reliability (α = 0.260) and discriminant validity issues. The negative coefficient should not be interpreted as evidence that ease of use reduces adoption intention but rather as a flag for measurement issues requiring attention in future research.

### 5.3. The Saudi Healthcare Context

As shown in Section 4.3.8, behavioral intention levels were moderate, indicating cautious readiness to adopt AI, with job displacement concerns remaining salient despite strong endorsement of collaborative models. These patterns suggested that acceptance was conditional on preserving professional roles and framing AI as augmenting rather than supplanting human expertise. This finding was particularly salient in the Saudi healthcare context. The emphasis on framing AI as an assistant rather than a replacement reflected cultural values around professional identity and job security in a society undergoing rapid economic transformation through Vision 2030. Healthcare professionals’ insistence on collaborative human-AI models suggested that successful adoption would require explicit reassurances about professional roles and career pathways.

The importance of Arabic language communication and culturally appropriate messaging emerged repeatedly in qualitative responses. Participants noted that AI systems and training materials were often available only in English, creating barriers for Arabic-speaking staff and patients. This linguistic consideration extended beyond simple translation to encompass cultural framing of AI benefits, risks, and ethical considerations in ways that resonated with Saudi values and norms.

Institutional readiness challenges were particularly acute in the Saudi context, where healthcare infrastructure varied substantially between large urban hospitals and smaller regional facilities. The mix of government, private, and teaching hospitals in the sample revealed disparities in IT infrastructure, training resources, and leadership commitment to AI adoption. These variations suggested that standardized national policies would need to accommodate different institutional capacities and readiness levels.

The regulatory and governance environment in Saudi healthcare remained in early stages of development regarding AI, as noted by multiple participants. The absence of clear liability frameworks, privacy regulations specific to AI, and oversight mechanisms created uncertainty that elevated perceived risks. This regulatory gap was not unique to Saudi Arabia but was particularly consequential in a context where healthcare professionals expressed strong expectations for ethical governance and accountability structures before committing to AI adoption.

### 5.4. Theoretical Contributions

This study made several theoretical contributions to the literature on AI acceptance in healthcare. First, it demonstrated the value of integrating ethical considerations from the MEAAM framework [9] into UTAUT-based acceptance models. The finding that Ethical Governance functioned as a distinct construct with unique predictive value, particularly through its mediating effects on trust and risk, suggested that standard technology acceptance models require augmentation when applied to medical AI, where ethical stakes are especially high.

Second, the study revealed a hierarchical relationship among acceptance determinants, where cognitive readiness (knowledge) preceded and enabled other factors such as perceived usefulness and social influence. This hierarchical structure challenged simpler models that treated all predictors as operating in parallel and suggested that adoption interventions should be sequenced strategically, with education preceding efforts to demonstrate benefits or mobilize social support.

Third, the study contributed to our understanding of AI acceptance in non-Western healthcare contexts. Much of the existing literature on medical AI adoption was in from North American and European settings [25]. This study demonstrated both universal patterns (the importance of knowledge, usefulness, and risk) and context-specific factors (cultural framing, linguistic considerations, regulatory gaps) that shaped acceptance in the Saudi healthcare environment. These findings underscored the need for culturally situated research on technology adoption rather than assuming universal applicability of Western-derived models.

It should be noted that the extended UTAUT-MEAAM model was not formally compared against the standard four-construct UTAUT baseline in this study. The incremental predictive value of incorporating ethical governance constructs therefore remains an empirical question for future model comparison research.

### 5.5. Research Limitations and Future Directions

Several limitations should be considered when interpreting these findings, each suggesting productive directions for future research. The cross-sectional design captured attitudes at a single point in time, precluding inferences about how perceptions evolved during actual AI implementation. Future longitudinal research should track acceptance across implementation stages, following individuals over time to understand how direct experience shapes perceptions and whether the dominance of knowledge as a predictor persists once healthcare professionals gain hands-on experience with AI systems.

The sample size of N = 119, while adequate for PLS-SEM analysis, limited statistical power for detecting smaller effects and prevented extensive subgroup analyses by profession, institutional type, or experience level. Future multi-institutional studies should therefore employ stratified random sampling to ensure balanced subgroup sizes. Gender moderation could be tested with a minimum of 60 participants per group; experience moderation could compare early-career versus experienced practitioners; and profession-based moderation could compare clinical staff versus administrative and student groups. Such analyses would reveal whether the dominance of Awareness/Knowledge as a predictor persists across demographic groups or whether acceptance mechanisms differ across professional contexts.

Although the sample represented diverse professional roles across government, private, and teaching hospitals, future research should employ stratified sampling to ensure more proportional representation across clinical and administrative functions, which would enable a more comprehensive examination of AI acceptance patterns across all healthcare stakeholder groups.

Measurement issues affected Performance Expectancy and Effort Expectancy, which showed low reliability (α = 0.382 and α = 0.260) and discriminant validity problems. Performance Expectancy showed low internal consistency and below-threshold AVE (0.462), suggesting item heterogeneity or conceptual overlap with other constructs. Effort Expectancy demonstrated similar problems (α = 0.260, AVE = 0.421). One possible reason for the low reliability and validity is the low number of items. The low alpha values can be attributed to a major limitation of Cronbach’s alpha: it assumes equal indicator loadings in the population (also referred to as tau-equivalence). The violation of this assumption manifests itself in lower reliability values than those produced by CR [26]. Additionally, Cronbach’s alpha values are reliant on the number of items in a construct; with a low number of items, the alpha values may be reduced further [29]. These two limitations could be strong contributors to the low alpha values in the study. Discriminant validity diagnostics revealed content overlap among Performance Expectancy, Effort Expectancy, Social Influence, and Trust, with two Fornell–Larcker violations and several HTMT values exceeding 0.85, indicating content overlap among these constructs that undermines their distinctiveness. These findings suggest the need for item refinement in future research, but did not invalidate the overall model, as the primary constructs of theoretical interest (AK, PR, Trust, FC) demonstrated strong psychometric properties. In particular, the significant path from Performance Expectancy to Behavioral Intention (β = +0.229, *p* < 0.05) should be interpreted with caution given the construct’s low internal consistency, as weak measurement reliability can introduce instability in structural path estimates. Future research should develop and validate measurement instruments specifically designed for medical AI contexts in Arabic-speaking populations, including cognitive interviewing to ensure item comprehension and confirmatory factor analysis to validate refined measurement models. Culturally validated instruments would enable more precise estimation of effects and clearer differentiation between related constructs.

The theoretical extension of UTAUT with MEAAM-derived ethical constructs was not formally tested against a baseline model in this study. Future studies should conduct nested model comparisons between the standard four-construct UTAUT and the extended model, reporting the change in R^2^ to demonstrate that incorporating ethical governance meaningfully improves predictive power beyond standard UTAUT.

The reliance on self-reported intentions rather than observed behavior represented another limitation, as intention-behavior gaps in technology adoption are well-documented. Future studies should track actual AI system usage through log data, direct observations, and experience sampling to validate whether acceptance predictors correspond to sustained use. Linking survey-measured intentions to objective usage metrics would identify additional factors facilitating or hindering the translation of positive attitudes into practice.

Geographic coverage was limited to accessible hospitals, potentially underrepresenting rural areas and smaller institutions. The purposive sampling strategy might have introduced selection bias toward individuals already interested in AI. Future research should employ probability sampling to ensure representative coverage across Saudi Arabia’s diverse healthcare landscape and conduct regional comparative studies to determine whether findings generalize across different institutional environments.

The rapid pace of AI development meant that perceptions reflected the capabilities existing at the time of data collection. Future research should continuously monitor acceptance as technologies advance and conduct technology-specific acceptance studies examining whether mechanisms differ across AI application domains such as diagnostic imaging, treatment recommendations, or patient-facing chatbots. Such domain-specific research would reveal whether the general acceptance model requires adaptation for different use cases.

Intervention studies testing acceptance-promoting strategies would provide actionable evidence for practitioners and policymakers. This study identified education, governance frameworks, and risk mitigation as key levers, but experimental designs are needed to evaluate which specific interventions produce the greatest gains. Randomized trials comparing training approaches, governance models, or communication strategies would generate evidence-based implementation guidelines.

Comparative research across different countries would illuminate which findings are universal versus context-specific. The Saudi healthcare environment features unique characteristics: rapid modernization, specific regulatory frameworks, Islamic cultural values, and Arabic language requirements. These characteristics may shape acceptance differently than in other settings. Cross-national comparisons would distinguish universal acceptance mechanisms from culturally situated factors and identify best practices that transfer across contexts.

Finally, future research should examine the interplay between individual acceptance and organizational implementation. This study focused on individual-level factors, but AI adoption is fundamentally organizational, involving institutional decision-making, resource allocation, and workflow redesign. Multi-level research integrating individual acceptance with organizational readiness would provide a complete picture of how AI becomes embedded in healthcare practice and identify organizational interventions that amplify individual-level acceptance effects.

## 6. Conclusions

This study investigated factors influencing AI acceptance among healthcare professionals in Saudi Arabia. Healthcare professionals demonstrated moderate AI awareness and cautious behavioral intention, with readiness existing but constrained by knowledge gaps and risk concerns. Knowledge and understanding of AI emerged as the primary driver of acceptance, followed by perceived usefulness and peer support. Perceived risks related to safety, privacy, and accountability functioned as the main barrier, while ethical governance expectations were very high. Trust showed minimal direct effect on intention, operating indirectly through its influence on risk perception. Several other hypothesized constructs, including Facilitating Conditions, Ethical Governance, Social Influence, Price Value, and Effort Expectancy, did not reach statistical significance, suggesting that their roles may be indirect, context-dependent, or detectable only with larger samples. Qualitative findings identified infrastructure gaps, regulatory ambiguities, and training deficiencies as major implementation barriers, with participants emphasizing the importance of framing AI as an assistant rather than a replacement for healthcare professionals.

The findings generate clear implications for AI implementation, prioritized by strength of evidence. First, education and training represent the highest-leverage intervention, as knowledge deficits currently constrain adoption despite positive attitudes. Second, risk mitigation and governance frameworks must be prioritized, as perceived safety, privacy, and accountability concerns remain the primary barrier. Third, leadership and peer endorsement should be actively mobilized, as qualitative evidence consistently highlighted management support as essential. Fourth, infrastructure and workflow integration should be addressed institutionally to remove practical barriers identified in qualitative responses.

Theoretically, this study provides preliminary evidence for the applicability of extended technology acceptance frameworks in non-Western healthcare contexts, suggesting that cognitive readiness may precede organizational enablers in early-stage AI adoption. The findings reveal context-specific patterns including professional identity concerns, linguistic and cultural adaptation needs, and the critical role of governance frameworks in reducing risk perceptions.

AI integration in Saudi healthcare is feasible but requires coordinated efforts across education, policy, institutional leadership, and technology development. By prioritizing knowledge development, transparent governance, and risk mitigation while ensuring AI systems augment rather than threaten professional roles, Saudi Arabia can advance healthcare AI adoption in ways that enhance clinical quality and efficiency while respecting cultural values and ethical principles.

## Figures and Tables

**Table 1:** Measurement Constructs, Item Count, and Sources Used in the Proposed Research Model.

Construct	Items (k)	Source
Awareness/Knowledge	5	Chen et al. [20]
Performance Expectancy	4	UTAUT [8]
Effort Expectancy	2	UTAUT [8]
Facilitating Conditions	2	UTAUT [8]
Social Influence	3	UTAUT [8]
Trust	4	Chen et al. [20]
Perceived Risk	2	Chen et al. [20]
Ethical Governance	3	MEAAM [9]
Price Value/ROI	3	UTAUT2 [21]
Behavioral Intention	1	UTAUT [8]

**Table 2:** Descriptive Statistics for Participant Demographic Characteristics (N = 119).

Demographic	Characteristic	Frequency (n)	Percentage (%)
Gender	Male	68	57.1%
	Female	51	42.9%
Age	18–24 Years	35	29.4%
	25–34 Years	41	34.5%
	35–44 Years	32	26.9%
	45–54 Years	9	7.6%
	More than 55 Years	2	1.7%
Profession	Physician	10	8.4%
	Nurse	32	26.9%
	Administrator	30	25.2%
	Student (Healthcare)	17	14.3%
	Other	30	26.9%
Experience	Less than 1 year	27	22.7%
	1–5 Years	32	26.9%
	6–10 Years	22	18.5%
	11–15 Years	19	16.0%
	More than 15 Years	19	16.0%
Institution Type	Government Hospital	63	52.9%
	Private Hospital	10	8.4%
	Other	46	38.7%

**Table 3:** Construct Descriptive Statistics and Reliability Metrics.

Construct	Mean	SD	α	AVE	CR
Awareness/Knowledge (AK)	3.79	1.07	0.818	0.849	0.918
Performance Expectancy (PE)	3.92	1.00	0.382	0.462	0.697
Effort Expectancy (EE)	3.98	0.96	0.260	0.421	0.674
Facilitating Conditions (FC)	3.90	0.98	0.799	0.507	0.861
Social Influence (SI)	4.02	0.93	0.587	0.711	0.831
Trust	4.12	0.85	0.704	0.635	0.837
Perceived Risk (PR)	3.92	1.02	0.732	0.791	0.883
Ethical Governance (EG)	4.21	0.82	0.978	0.936	0.978
Price Value/ROI (PV)	3.73	1.05	0.638	0.735	0.847
Behavioral Intention (BI)	3.48	1.23	—	—	—

Note: α = Cronbach’s alpha; AVE = Average Variance Extracted; CR = Composite Reliability. Behavioral Intention was measured with a single item following established UTAUT precedent for bounded technology adoption behaviors; this choice is discussed in Section 3.4.1.

**Table 4:** PLS-SEM Path Coefficients to Behavioral Intention.

Path	β	SE	t-Value	*p*-Value
Awareness/Knowledge → BI	+0.505	0.082	6.16	<0.001
Performance Expectancy → BI	+0.229	0.091	2.52	<0.05
Social Influence → BI	+0.123	0.089	1.38	0.168
Ethical Governance → BI	+0.094	0.078	1.21	0.227
Facilitating Conditions → BI	+0.077	0.085	0.91	0.363
Trust → BI	+0.019	0.093	0.20	0.841
Price Value/ROI → BI	−0.003	0.082	0.04	0.968
Effort Expectancy → BI	−0.083	0.088	0.94	0.347
Perceived Risk → BI	−0.185	0.086	2.15	<0.05
Model fit: *R*^2^ (BI)	0.598			

Note: β = standardized path coefficient; SE = standard error; BI = Behavioral Intention. Significance was assessed via bootstrapping with 5000 subsamples and bias-corrected 95% confidence intervals.

## Data Availability

The data presented in this study are openly available in the Center for Open Science at https://osf.io/vbqjs (accessed on 1 June 2026).

## Abbreviations

The following abbreviations are used in this manuscript:

CDSSs	Clinical Decision Support Systems
CR	Composite Reliability
PLS-SEM	Partial Least Squares Structural Equation Modeling
UTAUT	Unified Theory of Acceptance and Use of Technology
α	Cronbach’s Alpha (Internal Consistency Reliability)
β	Beta Coefficient (Standardized Path Coefficient)

## Appendix A

This appendix presents detailed frequency distributions for all survey items. Responses were given using a 5-point Likert scale (1 = Strongly Disagree, 5 = Strongly Agree). Sample size: N = 119.

**Table A1:** Awareness and Knowledge Items—Response Frequencies.

Item	Statement	Strongly Disagree	Disagree	Neutral	Agree	Strongly Agree
AK1	General AI knowledge	1 (0.8%)	13 (10.9%)	27 (22.7%)	44 (37.0%)	34 (28.6%)
AK2	AI in medical imaging	5 (4.2%)	16 (13.4%)	22 (18.5%)	47 (39.5%)	29 (24.4%)
AK3	CDSS awareness	3 (2.5%)	15 (12.6%)	23 (19.3%)	44 (37.0%)	34 (28.6%)
AK4	Remote monitoring	4 (3.4%)	17 (14.3%)	26 (21.8%)	37 (31.1%)	35 (29.4%)
AK5	Virtual assistants	4 (3.4%)	16 (13.4%)	22 (18.5%)	39 (32.8%)	38 (31.9%)

Note: AK1: I have a general knowledge of AI utilization in the health sector. AK2: I am aware of AI applications in medical image analysis (e.g., X-ray, MRI, CT scans). AK3: I am aware of Clinical Decision Support Systems (CDSS) that suggest diagnosis and treatment options. AK4: I am aware of AI-powered remote patient monitoring systems. AK5: I know virtual assistants or chatbots that provide healthcare consultations. Mean = 3.79, SD = 1.07, Cronbach’s α = 0.818.

**Table A2:** Performance Expectancy Items—Response Frequencies.

Item	Statement	Strongly Disagree	Disagree	Neutral	Agree	Strongly Agree
PE1	AI improves diagnosis precision	1 (0.8%)	6 (5.0%)	23 (19.3%)	41 (34.5%)	48 (40.3%)
PE2	AI reduces human mistakes	0 (0.0%)	4 (3.4%)	27 (22.7%)	42 (35.3%)	46 (38.7%)
PE3	AI helps accurate decisions.	0 (0.0%)	8 (6.7%)	34 (28.6%)	43 (36.1%)	34 (28.6%)
PE4	AI analytics improve efficiency	0 (0.0%)	4 (3.4%)	25 (21.0%)	41 (34.5%)	49 (41.2%)

Note: PE1: I believe AI can improve the precision of diagnosis in my clinical practice. PE2: The application of AI reduces the likelihood of human mistakes. PE3: AI helps accurate decision-making in the healthcare sector. PE4: AI-driven big data analytics make the distribution of healthcare resources more efficient. Mean = 3.92, SD = 1.00, Cronbach’s α = 0.382.

**Table A3:** Effort Expectancy Items—Response Frequencies.

Item	Statement	Strongly Disagree	Disagree	Neutral	Agree	Strongly Agree
EE1	Easy to learn AI systems	1 (0.8%)	4 (3.4%)	27 (22.7%)	46 (38.7%)	41 (34.5%)
EE2	Can handle surgical robots	5 (4.2%)	7 (5.9%)	31 (26.1%)	41 (34.5%)	35 (29.4%)

Note: EE1: I find it easy to learn how to use AI-based healthcare systems. EE2: I can handle AI-assisted surgical robots after receiving proper training. Mean = 3.98, SD = 0.96, Cronbach’s α = 0.260.

**Table A4:** Facilitating Conditions Items—Response Frequencies.

Item	Statement	Strongly Disagree	Disagree	Neutral	Agree	Strongly Agree
FC1	Sufficient training and support	6 (5.0%)	28 (23.5%)	33 (27.7%)	25 (21.0%)	27 (22.7%)
FC2	Sufficient IT infrastructure	2 (1.7%)	12 (10.1%)	37 (31.1%)	39 (32.8%)	29 (24.4%)

Note: FC1: The hospital I work with offers sufficient training and support for AI implementation. FC2: The hospital’s IT infrastructure is sufficient for AI integration. Mean = 3.90, SD = 0.98, Cronbach’s α = 0.799.

**Table A5:** Social Influence Items—Response Frequencies.

Item	Statement	Strongly Disagree	Disagree	Neutral	Agree	Strongly Agree
SI1	Colleague encouragement	1 (0.8%)	2 (1.7%)	23 (19.3%)	45 (37.8%)	48 (40.3%)
SI2	Management support	0 (0.0%)	3 (2.5%)	15 (12.6%)	44 (37.0%)	57 (47.9%)
SI3	Social perceptions	3 (2.5%)	9 (7.6%)	23 (19.3%)	47 (39.5%)	37 (31.1%)

Note: SI1: Colleague encouragement increases my willingness to adopt AI. SI2: Successful AI implementation depends on senior management support. SI3: Social perceptions (patient opinions, public acceptance) influence my willingness to adopt AI. Mean = 4.02, SD = 0.93, Cronbach’s α = 0.587.

**Table A6:** Trust Items—Response Frequencies.

Item	Statement	Strongly Disagree	Disagree	Neutral	Agree	Strongly Agree
T1	Trust AI recommendations	1 (0.8%)	3 (2.5%)	35 (29.4%)	50 (42.0%)	30 (25.2%)
T2	Concern in high-risk cases	1 (0.8%)	10 (8.4%)	27 (22.7%)	41 (34.5%)	40 (33.6%)
T3	Data privacy concerns	4 (3.4%)	11 (9.2%)	25 (21.0%)	41 (34.5%)	38 (31.9%)
T4	AI enhances security	2 (1.7%)	5 (4.2%)	27 (22.7%)	48 (40.3%)	37 (31.1%)

Note: T1: I trust the recommendations provided by AI-powered clinical decision-making systems. T2: I’m concerned about relying on AI in high-risk cases (e.g., cancer diagnosis, major surgery). T3: I worry about patient data privacy and confidentiality in AI systems. T4: I recognize AI’s potential to enhance data security through encryption and access controls. Mean = 4.12, SD = 0.85, Cronbach’s α = 0.704.

**Table A7:** Perceived Risk Items—Response Frequencies.

Item	Statement	Strongly Disagree	Disagree	Neutral	Agree	Strongly Agree
PR1	Legal accountability concerns	1 (0.8%)	8 (6.7%)	23 (19.3%)	43 (36.1%)	44 (37.0%)
PR2	AI error detection concerns	4 (3.4%)	21 (17.6%)	26 (21.8%)	31 (26.1%)	37 (31.1%)

Note: PR1: I worry about legal accountability for medical errors caused by AI systems. PR2: I’m concerned about AI’s ability to detect and prevent human errors in healthcare. Mean = 3.92, SD = 1.02, Cronbach’s α = 0.732.

**Table A8:** Ethical Governance Items—Response Frequencies.

Item	Statement	Strongly Disagree	Disagree	Neutral	Agree	Strongly Agree
EG1	Explainable and transparent	1 (0.8%)	1 (0.8%)	21 (17.6%)	42 (35.3%)	54 (45.4%)
EG2	Fairness and no bias	1 (0.8%)	2 (1.7%)	22 (18.5%)	47 (39.5%)	47 (39.5%)
EG3	Ethical guidelines	0 (0.0%)	6 (5.0%)	15 (12.6%)	43 (36.1%)	55 (46.2%)

Note: EG1: I believe that AI systems in medicine should be explainable and transparent. EG2: Fairness and lack of bias contribute to my readiness to accept AI decision-making. EG3: Clear ethical guidelines and accountability structures increase my trust toward AI adoption. Mean = 4.21, SD = 0.83.

**Table A9:** Price Value and ROI Items—Response Frequencies.

Item	Statement	Strongly Disagree	Disagree	Neutral	Agree	Strongly Agree
PV1	Positive ROI required	3 (2.5%)	6 (5.0%)	25 (21.0%)	38 (31.9%)	47 (39.5%)
PV2	Limited by funding	2 (1.7%)	22 (18.5%)	41 (34.5%)	29 (24.4%)	25 (21.0%)
PV3	Incentives encourage	0 (0.0%)	2 (1.7%)	32 (26.9%)	31 (26.1%)	54 (45.4%)

Note: PV1: Clear financial justification (positive ROI) should be proven before adopting AI. PV2: AI adoption in my workplace is limited by insufficient funding. PV3: Financial incentives or government support would encourage greater AI acceptance. Mean = 3.73, SD = 1.05, Cronbach’s α = 0.638.

**Table A10:** Behavioral Intention and Collaboration Items—Response Frequencies.

Item	Statement	Strongly Disagree	Disagree	Neutral	Agree	Strongly Agree
BI1	AI as helping tool	0 (0.0%)	2 (1.7%)	14 (11.8%)	34 (28.6%)	69 (58.0%)
BI2	Combining AI and professionals	1 (0.8%)	1 (0.8%)	15 (12.6%)	45 (37.8%)	57 (47.9%)
BI3	Replacement worries	8 (6.7%)	21 (17.6%)	26 (21.8%)	34 (28.6%)	30 (25.2%)

Note: BI1: AI should be employed as a helping tool to assist physicians rather than replace them. BI2: Combining AI tools with healthcare professionals is key to achieving success in medicine. BI3: Worries that AI might replace human experts reduce my willingness to use AI. Mean = 3.48, SD = 1.23.

**Table A11:** Open-Ended Question Response Rates.

Topic	Responses
Q1. What obstacles prevent AI implementation in Saudi hospitals?	69 (58.0%)
Q2. What opportunities do you perceive from AI to improve healthcare in Saudi Arabia?	60 (50.4%)
Q3. What recommendations would raise the level of AI acceptance (ethical and organizational)?	54 (45.4%)

Note: N = 119 total respondents. Percentages indicate proportion who provided substantive responses.

**Table A12:** AI Acceptance and Implementation Checklist for Saudi Healthcare Institutions.

Step	Category	Action Item
1	Assessment	Identify current AI tools and applications in use (CDSS, imaging, triage, etc.)
2	Needs Analysis	Survey staff to assess knowledge gaps and training needs regarding AI.
3	Infrastructure Readiness	Evaluate IT infrastructure and data interoperability for AI integration.
4	Tool Selection	Select AI systems aligned with clinical needs, budget, and regulatory requirements.
5	Ethical Governance	Develop or update guidelines for explainability, fairness, and legal accountability.
6	Data Security & Privacy	Ensure AI use complies with MOH and national privacy standards.
7	Phased Implementation	Pilot AI tools in select departments with clear evaluation criteria.
8	Staff Training	Deliver initial and ongoing AI training programs for all relevant staff.
9	Interdisciplinary Teams	Form teams combining clinical, IT, and administrative expertise.
10	Trust Building	Communicate benefits, limitations, and responsibilities of AI to staff/patients.
11	Monitoring & Evaluation	Establish dashboards and KPIs for tracking clinical and operational impact.
12	Feedback Mechanisms	Collect and address staff feedback and incident reports regarding AI use.
13	Continuous Improvement	Review outcomes regularly; update strategies based on feedback and evidence.
14	Future Readiness	Stay informed on emerging AI trends, tools, and regulatory changes.

This checklist should be used as a living document, reviewed quarterly during implementation and updated based on organizational experience and evolving best practices.
